# Sequence analysis of the GP5 protein of porcine reproductive and respiratory syndrome virus in Vietnam from 2007 to 2023

**DOI:** 10.3389/fmicb.2024.1475208

**Published:** 2024-10-01

**Authors:** Gan Li, Yilong Li, Cuihua He, Xiyu Liu, Chen Lv, Kexin Liu, Xingang Yu, Mengmeng Zhao

**Affiliations:** Guangdong Provincial Key Laboratory of Animal Molecular Design and Precise Breeding, School of Animal Science and Technology, Foshan University, Foshan, China

**Keywords:** PRRSV, *GP5* gene, homology, phylogeny, genetic variation

## Abstract

**Introduction:**

Porcine reproductive and respiratory syndrome virus (PRRSV) is the causative agent 13 of porcine reproductive and respiratory syndrome (PRRS), which is one of the most economically 14 devastating viruses in the Vietnamese swine industry.

**Methods:**

With a view toward determining the 15 genetic variation among PRRSV strains in Vietnam, we examined 271 PRRSV *GP5* protein 16 sequences obtained from strains isolated in Vietnam from 2007 to 2023, for which we constructed 17 phylogenetic trees. Additionally, a collection of 52 PRRSV-1 strains and 80 PRRSV-2 strains 18 isolated in different years were specifically selected for nucleotide and amino acid homology analysis 19 and amino acid sequence alignment.

**Results:**

The results revealed 76.1%–100.0% nucleotide and 20 75.2%–100.0% amino acid homologies for the PRRSV-1 *GP5* gene, and 81.8%–100.0% nucleotide 21 and 81.1%–100.0% amino acid homologies for the PRRSV-2 *GP5* gene. Amino acid mutation sites 22 in PRRSV-2 were found to be primarily distributed in the signal peptide region, antigenic sites, two 23 T-cell antigen regions, two highly variable regions (HVRs), and in the vicinity of the neutralizing 24 epitope, with a deletion mutation occurring in the neutralizing epitope, whereas amino acid mutations 25 in the PRRSV-1 sequences were found to occur predominantly in two T-cell epitopes. Genetic 26 analysis revealed that PRRSV-1 strains in Vietnam are of subtype 1 (Global), whereas PRRSV-2 27 strains are categorized into sublineages L1A, L5A, and L8E, with L8E being the predominantly 28 prevalent strain at present. Recombination analyses indicated that no significant recombination 29 events have occurred in any of the assessed 271 Vietnamese PRRSV strains.

**Discussion:**

Our 30 analyses of 271 Vietnamese PRRSV strains have yielded valuable insights regarding the 31 epidemiological trends and genetic dynamics of PRRSV in Vietnam, and will provide a theoretical 32 basis for formulating prevention and control measures for PRRS and the development of PRRS 33 vaccines.

## 1 Introduction

The porcine reproductive and respiratory syndrome virus (PRRSV), the causal pathogen of porcine reproductive and respiratory syndrome (PRRS), primarily infects swine herds, causing reproductive disorders in pregnant sows, including miscarriage, the occurrence of stillbirths and mummified fetuses, as well as respiratory diseases in pigs of all ages (Sha et al., 2023). Since the initial isolation of PRRSV in Europe and the Americas in the early 1990s (Collins et al., 1992; Wensvoort et al., 1991), PRRS has become globally prevalent in many countries. In 2006, an outbreak of highly pathogenic porcine reproductive and respiratory syndrome (HP-PRRS), characterized by high fever and mortality in pigs of all ages, occurred in China and spread rapidly to many Asian countries (Guo et al., 2013). The incidence of HP-PRRS ranged from 50 to 100%, with mortality rates varying between 20 and 100%, resulting in the culling of approximately 40 million pigs in China in 2006–2007 (An et al., 2011), causing huge economic losses to the swine industry. However, given a range of complicating factors, including genetic diversity, immunosuppression, and secondary infections, the complete eradication of PRRS in the global swine industry presents significant challenge (Cai et al., 2023). Consequently, PRRS continues to pose a serious economic threat to the global swine industry.

PRRSV, a member of the viral genus *Arterivirus*, within the family *Arteriviridae* and order *Nidovirales*, is an enveloped, single-stranded positive-sense RNA virus (Adams et al., 2017). The PRRSV genome is approximately 15 kb in length and encodes 11 open reading frames (ORFs) (Zhang et al., 2023). ORF1a and ORF1b encode two polyproteins, namely, pp1a and pp1ab, respectively, which are subsequently cleaved by protein hydrolases to generate 16 non-structural proteins, whereas ORF2a, ORF2b, ORFs3–7, and ORF5a encode eight corresponding structural proteins (Chen et al., 2023). At present, PRRSV is divided into two distinct species, *Betaarterivirus suid* 1 (PRRSV-1) and *Betaarterivirus suid* 2 (PRRSV-2), which have approximately 60% nucleotide homology (Brinton et al., 2021; Lu et al., 2014). Among the viral proteins, the GP5 protein encoded by ORF5 is characterized by the highest structural diversity between PRRSV-1 and PRRSV-2 strains. At the amino acid level, the homology of PRRSV-1 and PRRSV-2 GP5 is between 50 and 55%, and this protein is thus commonly used in genetic sequence analysis (Lunney et al., 2010; Zhao et al., 2022). On the basis of ORF5 sequences, PRRSV-1 can be classified into four subtypes, namely, subtype 1 (Global), subtype 1 (Russia), subtype 2, and subtype 3, whereas PRRSV-2 can be classified into nine lineages and 37 sublineages (Li et al., 2022; Shi et al., 2010). However, Yim-Im et al. (2023) refined a phylogenetic classification system based on the PRRSV-2 ORF5, reclassifying PRRSV-2 into 11 lineages (L1-L11), 21 sublineages (L1A-L1F, L1H-L1J, L5A-L5B, L8A-L8E, and L9A-L9E), as well as the 5 taxa within L1C (L1C.1-L1C.5). Although partial sequences of PRRSV-1 strains isolated in Vietnam have been uploaded to the GenBank database, to the best of our knowledge, there have been no systematic studies on PRRSV-1 conducted to date, unlike PRRSV-2, for which there have been a number of studies. In 2007, an outbreak of highly pathogenic PRRS (HP-PRRS) was detected in Vietnam, which spread rapidly nationwide (Lee et al., 2019a). In the same year, Feng et al. (2008) isolated suspected PRRSV strains for the first time, and on the basis of bioinformatics analysis, revealed that these were strains of the PRRSV-2 species, with a 99% nucleotide homology to PRRSV-2 strains that were prevalent in China in 2006. However, as early as 2002, Kamakawa et al. (2006) had already detected PRRSV on free-range pig farms in Vietnam, thereby indicating that PRRS was prevalent in Vietnam prior to 2007. Do et al. (2016) found that among PRRSV field strains isolated from 2007 to 2015, all were of PRRSV-2 sub-lineages 8.7 and 5.1. Whereas for strains isolated in 2021, Nguyen et al. (2022) detected those of lineage 8, sublineage 8.7, lineage 1, and sublineage 1.4.

Since the 2007 PRRS outbreak, although numerous PRRS vaccines had been introduced in Vietnam (Lee et al., 2019b), until 2021, many swine farms in Vietnam on which pigs had for long been vaccinated with PRRS attenuated vaccines continued to experience PRRS outbreaks, and it was accordingly speculated that mutations in the GP5 were reducing the protective efficacy of these vaccines (Nguyen et al., 2022). To confirm this supposition and gain further insights into the genetic variations of the PRRSV *GP5* gene in Vietnam from 2007 to 2023, we obtained 344 PRRSV GP5 sequences from the GenBank database and used these to construct phylogenetic trees. Among these sequences, we selected 52 PRRSV-1 and 80 PRRSV-2 GP5 sequences for amino acid and nucleotide homology analyses and amino acid sequence alignment, along with analyses of potential recombination events. Our findings in this study will provide a theoretical foundation for understanding future epidemiological trends in PRRS and for the development of vaccine-based prevention and control measures in Vietnam.

## 2 Materials and methods

### 2.1 Dataset used for sequence analysis

The GP5 sequences of 344 PRRSV isolates were obtained from the GenBank, NCBI database. Given the limited number of sequences of Vietnamese PRRSV-1 strains deposited in GenBank, we selected 52 PRRSV-1 strains (25 from Vietnam; 13 from China; 6 from the United States; 1 from Spain; 5 from Russia; 2 from Belarus) (Table 1) and 292 PRRSV-2 strains (246 from Vietnam; 21 from China; 25 from the United States) (Supplementary Table S1). Moreover, as the earliest database accession from Vietnam is a 2007 isolate, we selected PRRSV strains that were isolated from 2007 to 2023. To ensure the comprehensiveness of our genetic dynamics analysis of PRRSV in Vietnam, all 52 selected PRRSV-1 strains were analyzed for genetic variation (Table 1), whereas 80 strains of different lineages (including classical, vaccine and epidemic strains) isolated in different years were selected from the 292 PRRSV-2 GP5 sequences (Table 2).

**Table 1:** The 52 PRRSV-1 GP5 reference sequences.

Year	Area	Strain	Genbank accession number
2016	Viet Nam	EuroViet-01	MG251834
2016	Viet Nam	EuroViet-02	MG251833
2016	Viet Nam	EuroViet-03	MG251835
2021	Viet Nam	PRRS-EU-VN01	OR135689
2021	Viet Nam	PRRS-EU-VN02	OR135690
2021	Viet Nam	PRRS-EU-VN03	OR135691
2021	Viet Nam	PRRS-EU-VN04	OR135692
2021	Viet Nam	PRRS-EU-VN05	OR135693
2022	Viet Nam	PRRS-EU-VN06	OR135694
2022	Viet Nam	PRRS-EU-VN07	OR135695
2022	Viet Nam	PRRS-EU-VN08	OR135696
2022	Viet Nam	PRRS-EU-VN09	OR135697
2022	Viet Nam	PRRS-EU-VN010	OR135698
2022	Viet Nam	PRRS-EU-VN11	OR135699
2022	Viet Nam	PRRS-EU-VN12	OR135700
2022	Viet Nam	PRRS-EU-VN13	OR135701
2022	Viet Nam	PRRS-EU-VN14	OR135702
2022	Viet Nam	PRRS-EU-VN15	OR135703
2022	Viet Nam	PRRS-EU-VN16	OR135704
2022	Viet Nam	PRRS-EU-VN17	OR135705
2022	Viet Nam	VNUA/PRRSV/HY4/22	OR800828
2023	Viet Nam	PRRS-EU-VN18	OR135706
2023	Viet Nam	PRRS-EU-VN19	OR135707
2023	Viet Nam	PRRS-EU-VN20	OR135708
2023	Viet Nam	VNUA/PRRSV/HY1/21	OM860456
2007	China	HKEU16	EU076704
2006	China	BJEU06-1	GU047344
2006	China	FJ0603	HM114313
2009	China	NMEU09-1	GU047345
2011	China	NVDC-NM3	KC492505
2015	China	FJEU13	KP860912
2016	China	HENZMD-10	KY363382
2018	China	EUGDHD2018	MK639926
2018	China	KZ2018	MN550991
2020	China	TZJ660_HN	OQ920881
2020	China	TZJ226	OP566682
2022	China	HBEU-328	OR636058
2023	China	SL-01	OQ871594
2003	USA	EuroPRRSV	AY366525
2003	USA	SD03-15_P83	KU131560
2004	USA	SDPRRS 04–48	AY749411
2004	USA	MN-04-09_EU	AY749397
2015	USA	USA/ISU78021/2015	MK359263
2016	USA	PRRSV1/USA/Lab6	MN175678
2009	Spain	Amervac PRRS	GU067771
2007	Russia	VR	EU071233
2013	Russia	WestSib13	KX668221
2016	Russia	Tyu16	MT008024
2007	Russia	RS	EU071230
2007	Russia	KZ-2	EU071239
2005	Belarus	Zad-1	DQ324694
2010	Belarus	SU1-Bel	KP889243

**Table 2:** The 80 PRRSV-2 GP5 reference sequences.

Year	Area	Strain	Genbank accession number
2007	Viet Nam	SRV07	JX512910
2008	Viet Nam	DN452	JQ860363
2009	Viet Nam	28SGVN	GU187016
2009	Viet Nam	BCL-PS 100	GU187014
2010	Viet Nam	10HuY	KF523293
2010	Viet Nam	Hanvet1.vn	KU842720
2010	Viet Nam	MB6	KM244761
2010	Viet Nam	BD.R1	JQ860381
2010	Viet Nam	HCMC-3336	HQ540646
2011	Viet Nam	NAVET/TG/PVR34	MZ934413
2011	Viet Nam	HUA-Viet.labPRRS1	AB856283
2012	Viet Nam	CT.C1	JQ860382
2012	Viet Nam	HG.RV2	JQ860391
2013	Viet Nam	MN1	KM244763
2013	Viet Nam	NamDinh-01	LC361112
2013	Viet Nam	D1/HCM1/VN_2013	KR261789
2014	Viet Nam	D8/DN3/VN_2014	KR261776
2014	Viet Nam	KTY-PRRS-08	LC192548
2015	Viet Nam	KTY_PRRS_02	LC102500
2015	Viet Nam	VN/NIVR-15	MZ158159
2015	Viet Nam	BN_10	LC105643
2016	Viet Nam	HUA/HP44	KY190024
2020	Viet Nam	P2581	MW366748
2021	Viet Nam	VN/HVBP3/2021	MZ218089
2021	Viet Nam	VN/HVBP4/2021	MZ218090
2021	Viet Nam	VN/HVDN2/2021	MZ218077
2021	Viet Nam	VNUA/PRRSV/PT2/21	OR800813
2021	Viet Nam	VN/HVDN1/2021	MZ218076
2021	Viet Nam	VNUA/PRRSV/DNai1/21	OR800803
2022	Viet Nam	VNUA/PRRSV/TH1/22	OR800824
2022	Viet Nam	VNUA/PRRSV/Hanoi1/22	OR800817
2022	Viet Nam	VNUA/PRRSV/HN1/22	OR800821
2023	Viet Nam	VNUA/PRRSV/TH1/23	OR800842
2023	Viet Nam	VNUA/PRRSV/TB2/23	OR800841
1996	China	BJ-4	AF331831
1996	China	CH-1a	AY032626
2006	China	JXA1	EF112445
2007	China	GD	EU109503
2008	China	CH-1R	EU807840
2008	China	WUH3	HM853673
2009	China	09HUB1	JF268682
2010	China	FS	JF796180
2012	China	TJM-F92	MN508255
2013	China	HeNan-A9	KJ546412
2014	China	SDZZ	KY373217
2015	China	15GD3	KX815409
2016	China	HNhx	KX766379
2017	China	GDZQ	KY488473
2018	China	HB18-41	MT268280
2019	China	SC/DJY	MT075480
2020	China	rJXA1-R	MT163314
2021	China	JS2021NADC34	MZ820388
2022	China	PRRSV-CH-SDLY27-2022	OP730529
2023	China	GD-7	OR711915
2023	China	TZJ3005	OR826313
1992	USA	VR-2332	EF536003
1993	USA	ATCC VR-2332	U87392
1996	USA	SDSU73	JN654458
1998	USA	RespPRRS MLV	AF066183
1999	USA	MLV RespPRRS/Repro	AF159149
2002	USA	P129	AF494042
2003	USA	JA142	AY424271
2004	USA	NVSL 97–7,895	AY545985
2005	USA	MN184A	DQ176019
2007	USA	PRRSV2/USA/LS12/2007	KU502673
2008	USA	NADC30	JN654459
2008	USA	NADC31	JN660150
2009	USA	PRRSV2/USA/Lab7	MT269876
2010	USA	IP178	MZ304150
2013	USA	IA/2013/ISU-1	MF326988
2014	USA	SDSU47	KT258009
2014	USA	IA/2014/NADC34	MF326985
2015	USA	A2MC2-P90	KU318406
2016	USA	NCV1	ON950548
2017	USA	NC_134/2017	ON844087
2018	USA	PRRSGard	OR293983
2018	USA	ISU1014_ZMAC	ON100575
2020	USA	NC20-1	OR805486
2021	USA	PF21	MW592736
2022	USA	USA/IN/04584GF/2022	OR669517

### 2.2 Phylogenetic analysis

The 344 selected PRRSV GP5 sequences were used to perform phylogenetic analysis, with phylogenetic trees being constructed using MEGA software (Version 11.0.13) based on the maximum likelihood (ML) (substitution model: Nucleotide; Model/Method: Tamura-Nei model; Rates among Sites: Gamma Distributed With Invariant Sites (G + I); ML Heuristic Method: Nearest-Neighbor-Interchange (NNI); Initial Tree for ML: Make initial tree automatically (Default - NJ/BioNJ)) and neighbor-joining (NJ) (substitution model: Nucleotide; Model/Method: p-distance; Substitutions to Include: d: Transitions + Transversions; Rates among Sites: Uniform Rates) methods with 100 and 1,000 bootstrap replicates, respectively. Default parameter values were utilized for other parameters. The phylogenetic tree data thus obtained were imported into online software (The Interactive Tree Of Life^1^) for subsequent sorting and editing.

### 2.3 GP5 nucleotide and amino acid homology analysis and sequence alignment

For analysis of PRRSV GP5 nucleotide and amino acid homologies, we used the GraphPad Prism (Version 9.0.0) and the Clustal W method in the MegaAlign function of DNASTAR software (Version 7.0). DNASTAR software was also used for amino acid sequence comparisons.

### 2.4 Recombination analysis

To facilitate preliminary identification of potential recombination events in the 344 assessed PRRSV GP5 sequences, we used the RDP, GENECONV, BootScan, MaxChi, Chimera, SiScan, and 3Seq algorithms in RDP software (Version 4.0). Strains for which four or more positive (+) results and *p* < 0.05 were obtained were considered recombinant strains.

## 3 Results

### 3.1 Phylogenetic analysis

**Figure 1:**
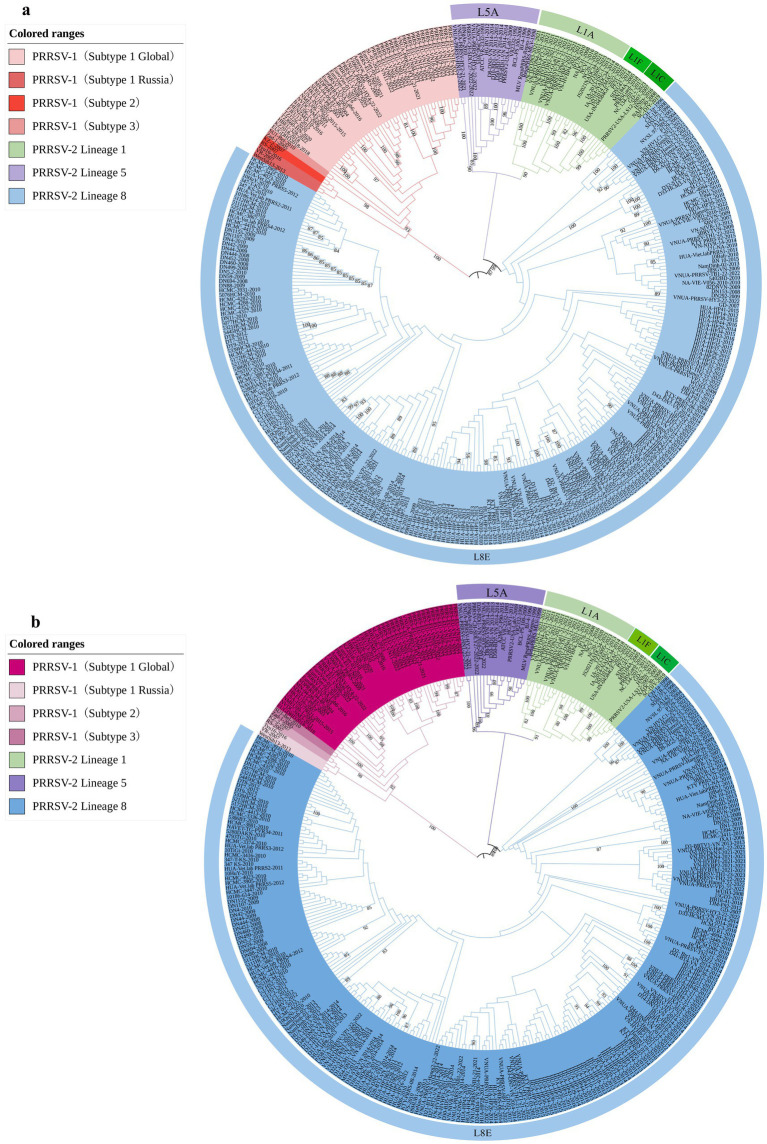
Phylogenetic analysis was conducted for 344 PRRSV GP5 sequences. **(A)** The construction of a phylogenetic tree was performed using the neighbor-joining method in MEGA software with 1,000 bootstrap replicates. **(B)** The construction of a phylogenetic tree was performed using the maximum likelihood method in MEGA software with 100 bootstrap replicates.

To determine the genetic variation of the PRRSV *GP5* gene in Vietnam, we analyzed the evolution of this viral gene by constructing phylogenetic trees using gene sequences for 52 PRRSV-1 and 80 PRRSV-2 strains, which were selected based on the availability of GP5 sequence information in the global PRRSV classification system and GenBank database. The results revealed that all PRRSV-1 strains prevalent in Vietnam are subtype 1 (Global) (Figure 1). Contrastingly, the PRRSV-2 strains were grouped into three lineages (1, 5, and 8), among which, lineage 8 strains were identified as being predominant in Vietnam. The most recent genetic classification of PRRSV-2 (Yim-Im et al., 2023) indicates that the lineage 1 strains in Vietnam were part of sublineage L1A, while the lineage 5 strains were classified as sublineage L5A and the lineage 8 strains were identified as sublineage L8E. PRRSV-1 subtype 1 (Global) strains were genetically distant from the subtype 1 (Russia) strains and exhibited a closer genetic relationship with subtype 3. PRRSV-2 L1A and L5A are the most closely related, while they are more distantly related to L8E. In addition, PRRSV-2 strains recently detected in Vietnam in 2023 were established to be lineage 8 strains.

### 3.2 Nucleotide similarity

**Figure 2:**
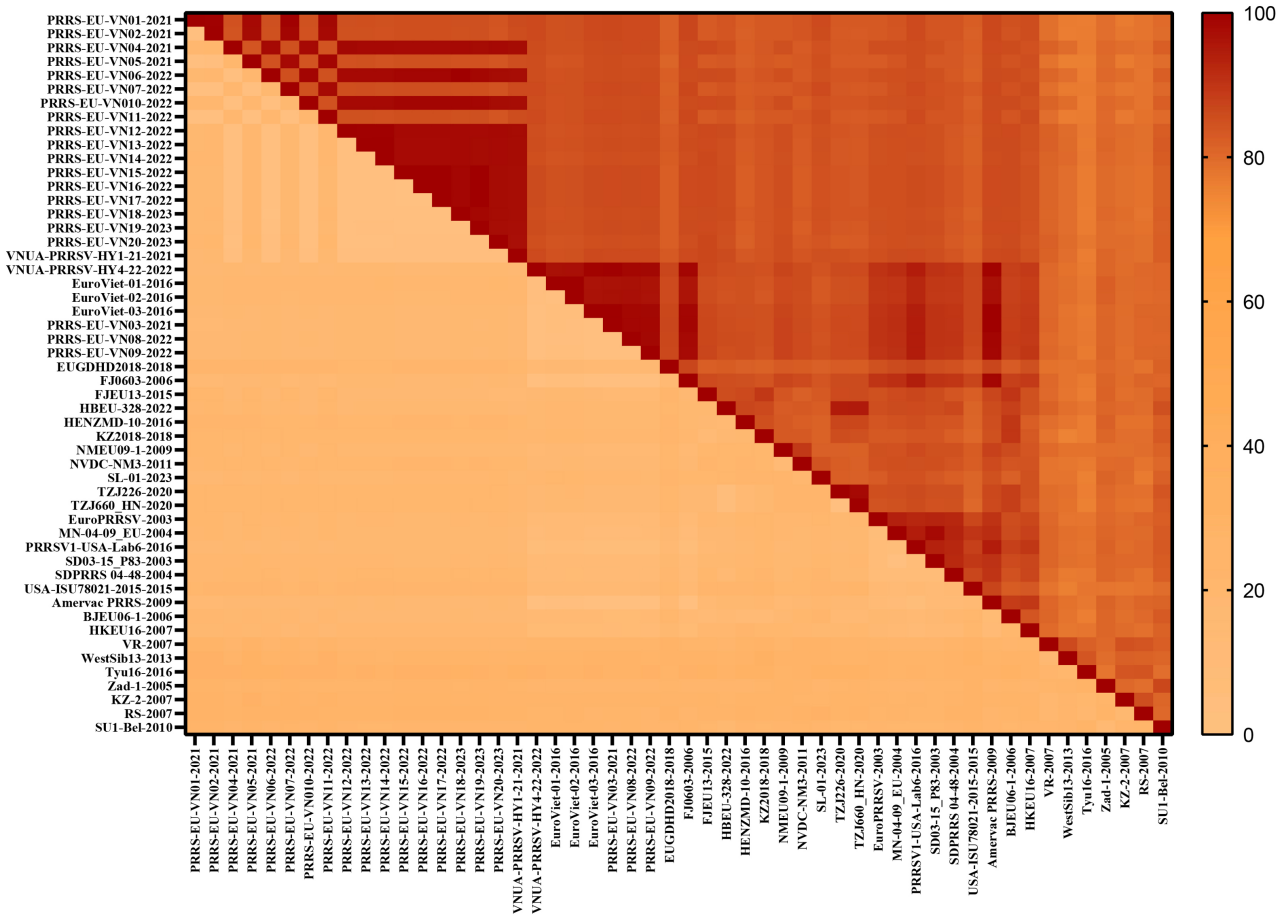
Analysis of the nucleotide homologies of 52 PRRSV-1 GP5 strains.

**Figure 3:**
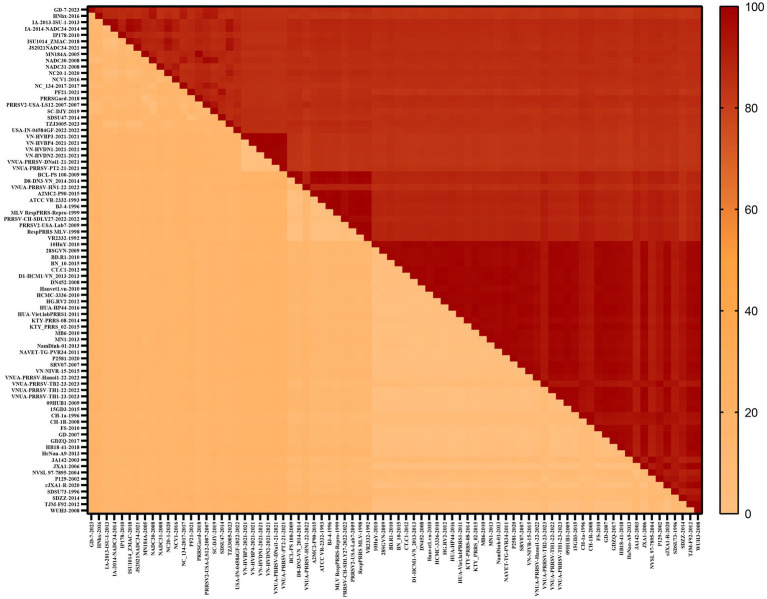
Analysis of the nucleotide homologies of 80 PRRSV-2 GP5 strains.

To gain a comprehensive understanding of the evolutionary genetic relationships among the different species and lineages of PRRSV, we performed nucleotide homology analyses for the PRRSV GP5 sequences. Analyses of the PRRSV-1 and PRRSV-2 GP5 sequences from Vietnam and other countries revealed nucleotide homologies 76.1–100.0% and 81.8–100.0%, respectively. Among the PRRSV-1 GP5 sequences (Figure 2; Supplementary Table S2), the lowest nucleotide homology (76.1%) was found between the strains of Vietnam and subtype 1 (Russia), whereas the PRRS-EU-VN06-2022 and PRRS-EU-VN18-2023 strains, and PRRS-EU-VN15-2022, PRRS-EU-VN16-2022, and PRRS-EU-VN17-2022 strains were characterized by the highest nucleotide homologies (100.0%). Among the PRRSV-2 GP5 sequences (Figure 3; Supplementary Table S3), the VNUA-PRRSV-DNai1-21–2021 and VNUA-PRRSV-HN1-22-2022 strains were found to have the lowest nucleotide homology (81.8%), whereas the VN-HVBP3-2021 and VN-HVBP4-2021 strains, the D1-HCM1-VN_2013 and MN1-2013 strains, and the HCMC-3336-2010 and NAVET-TG-PVR34-2011 strains had the highest nucleotide homologies (100.0%).

### 3.3 Amino acid sequence similarity

**Figure 4:**
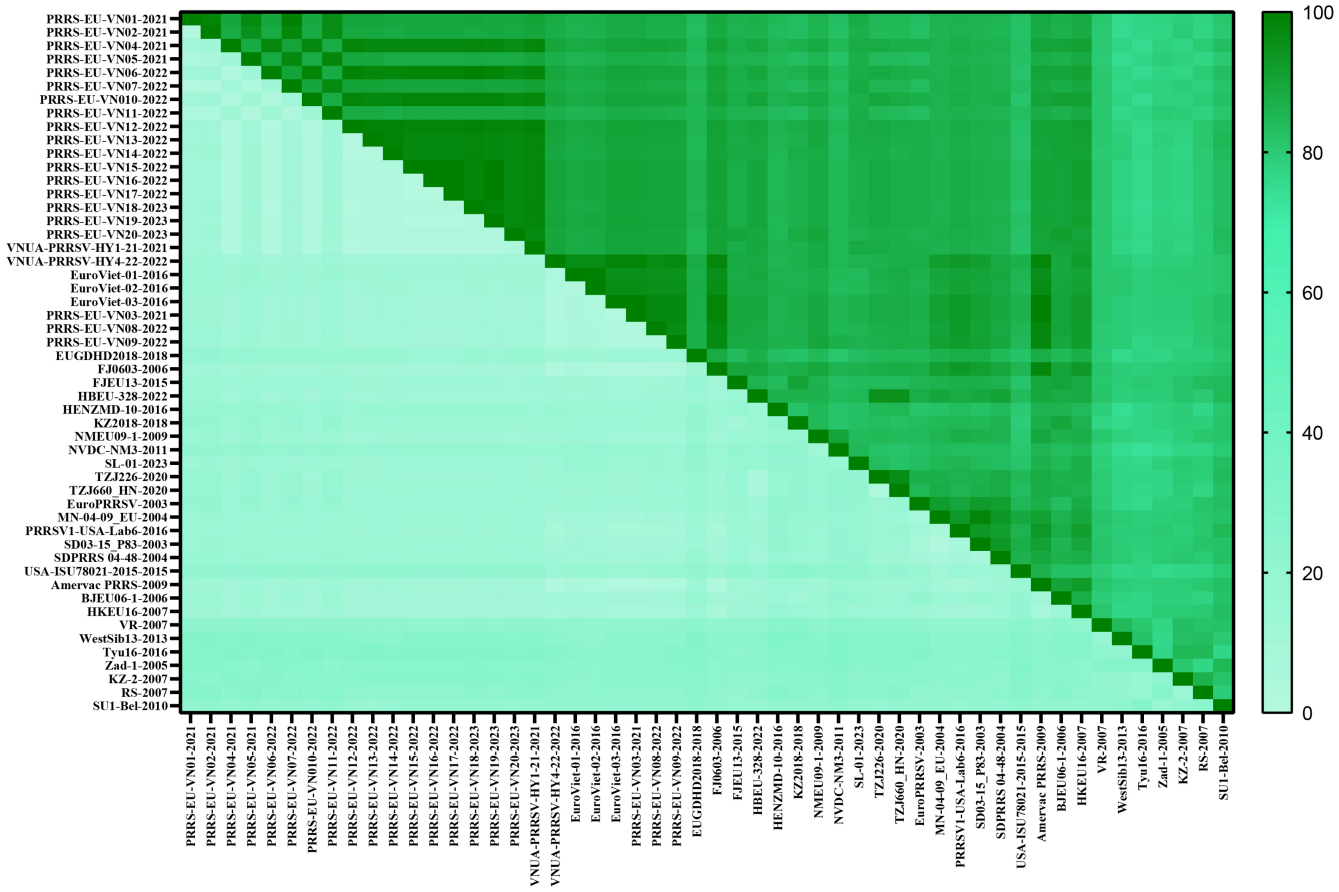
Analysis of the amino acid homologies of 52 PRRSV-1 GP5 strains.

**Figure 5:**
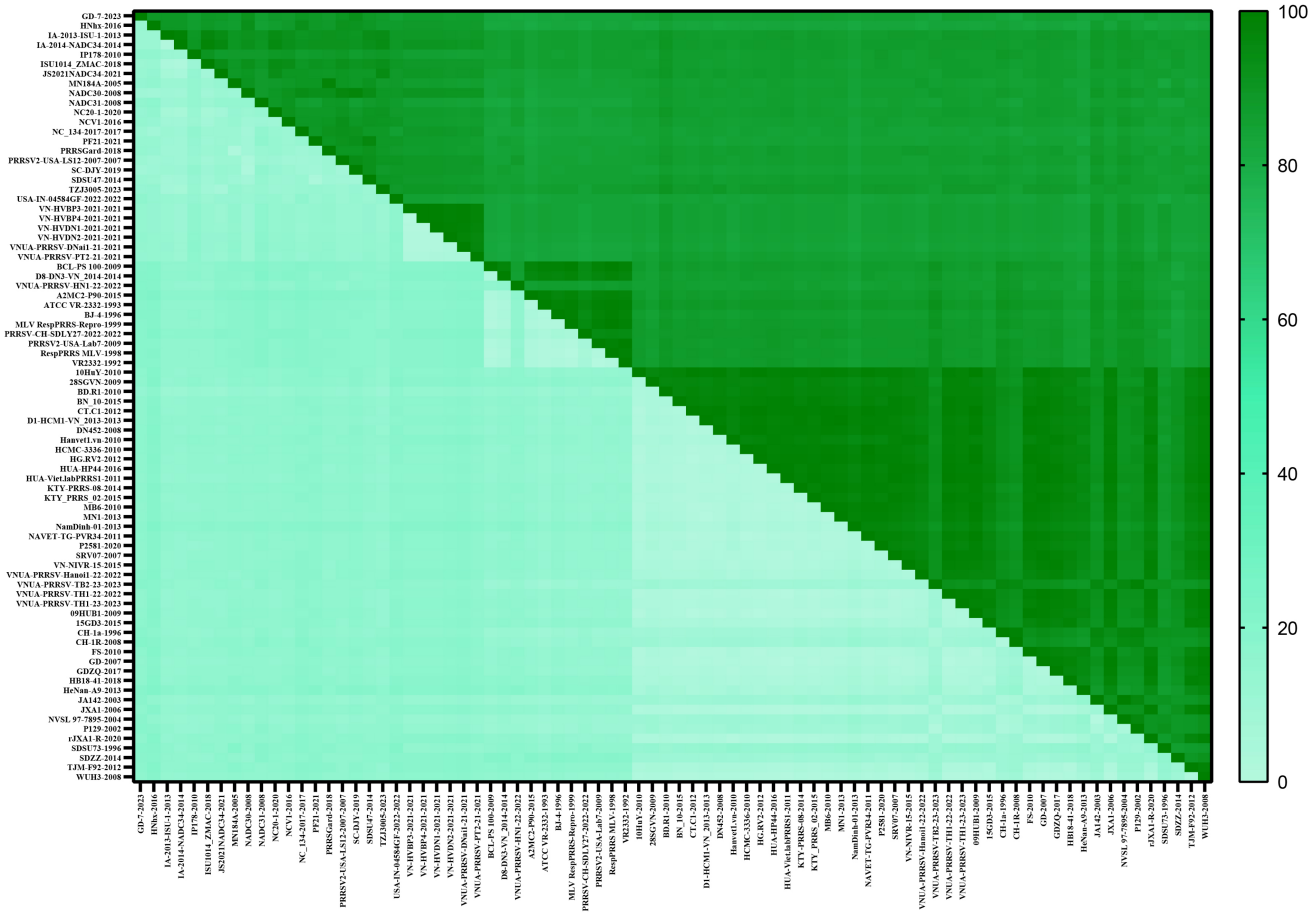
Analysis of the amino acid homologies of 80 PRRSV-2 GP5 strains.

Analysis of the amino acid homologies of PRRSV GP5 sequences revealed homologies of 75.2–100.0% and 81.1–100.0% for the *GP5* gene in PRRSV-1 and PRRSV-2 strains between Vietnam and other countries, respectively. Among the PRRSV-1 GP5 sequences (Figure 4; Supplementary Table S4), the lowest amino acid homology (75.2%) was detected between the strains of Vietnam and subtype 1 (Russia), whereas the highest homologies (100%) were between the PRRS-EU-VN03-2021 and EuroViet-03-2016 strains, PRRS-EU-VN06-2022 and PRRS-EU-VN18-2023 strains, and PRRS-EU-VN15-2022, PRRS-EU-VN16-2022, PRRS-EU-VN17-2022, and PRRS-EU-VN19-2023 strains. Among the 80 PRRSV-2 GP5 sequences (Figure 5; Supplementary Table S5), the HNhx-2016 and NamDinh-01–2013 strains, D8-DN3-VN_2014–2014 and NADC31-2008 strains had the lowest amino acid homology (81.1%), whereas the highest homologies (100%) were between the VN-HVBP3-2021 and VN-HVBP4-2021 strains, the D1-HCM1-VN_2013 and MN1-2013 strains, the CT.C1-2012, HG.RV2-2012 and 09HUB1-2009 strains, the HCMC-3336-2010 and NAVET-TG-PVR34-2011 strains, and the HUA-Viet.labPRRS1-2011 and VNUA-PRRSV-TH1-22-2022 strains.

### 3.4 Amino acid sequence alignment

**Figure 6:**
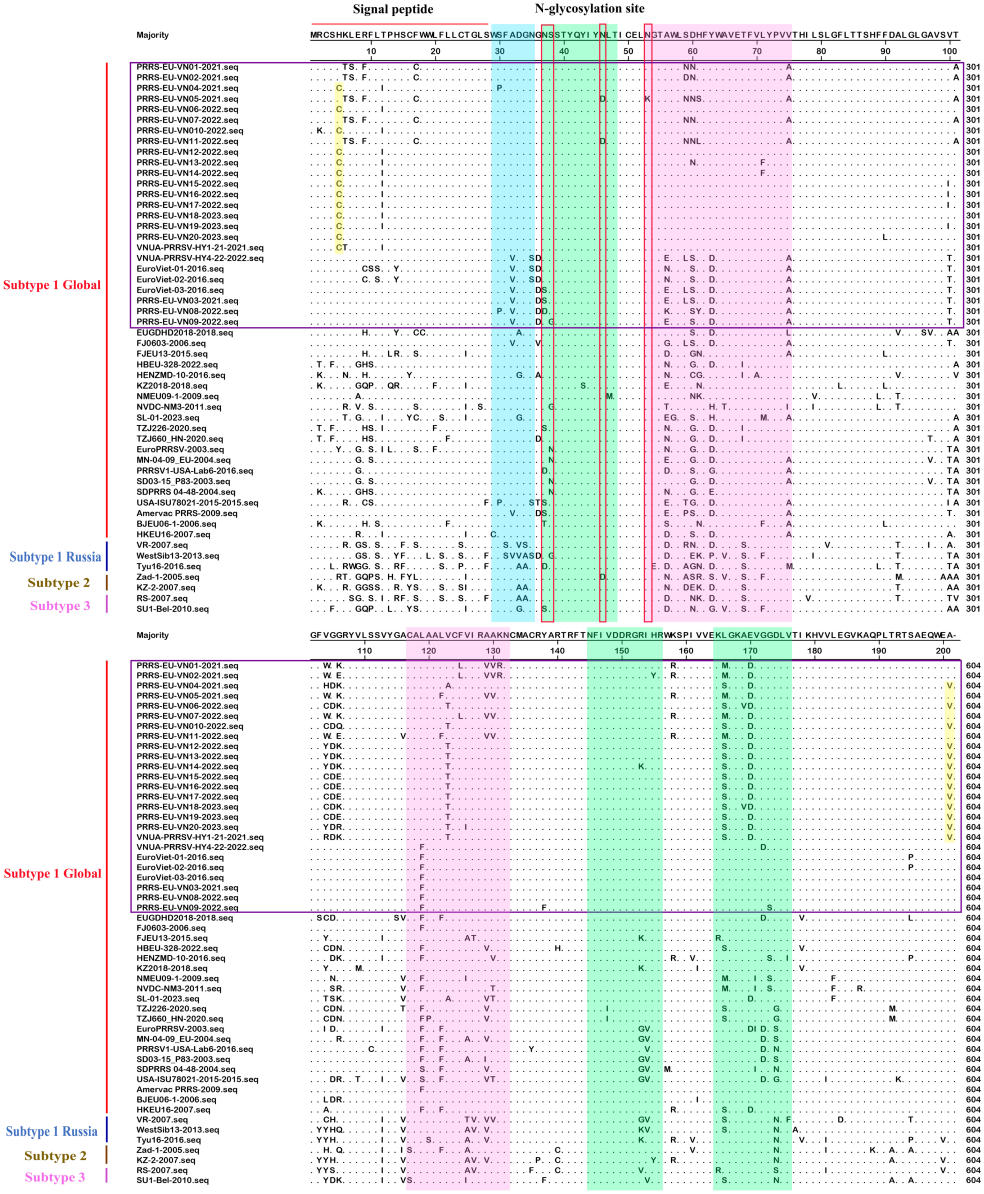
Alignment of 52 PRRSV-1 GP5 amino acid sequences. The Vietnamese strains are marked in purple. Potential neutralizing epitopes are marked. The Neutralizing epitope, B-cell epitope, T-cell epitope, and strain-specific mutations are represented by blue, green, pink, and yellow, respectively.

On the basis of our multiple alignments of the GP5 sequences of 52 PRRSV-1 and 80 PRRSV-2 strains, we established that whereas there have been no insertion mutations in any of the assessed PRRSV GP5 sequences, and a deletion mutation has occurred in Vietnamese PRRSV-2 strains (L1A). In the GP5 sequence of PRRSV-1 subtype 1 (Global) (Figure 6), the sites of amino acid mutation were found to be concentrated primarily in two T-cell epitopes, with four mutations being detected in its *N*-glycosylation site. Of these mutations, an N^37^S alteration has occurred in EuroViet-03-2016 and PRRS-EU-VN03-2021, an N^37^D alteration has occurred in PRRS-EU-VN08-2022, an S^38^G alteration has occurred in PRRS-EU-VN09-2022, an N^46^D alteration was found in PRRS-EU-VN05-2021 and PRRS-EU-VN11-2022, whereas a N^53^K transition was observed in PRRS-EU-VN05-2022. In addition, some Vietnamese strains were found to be characterized by two specific mutations (H^5^C and A^201^V), one of which was located at the signal peptide. These analyses also revealed that the GP5 sequence of PRRSV-1 strain in Vietnam has fewer mutation sites and a lower degree of variation.

**Figure 7:**
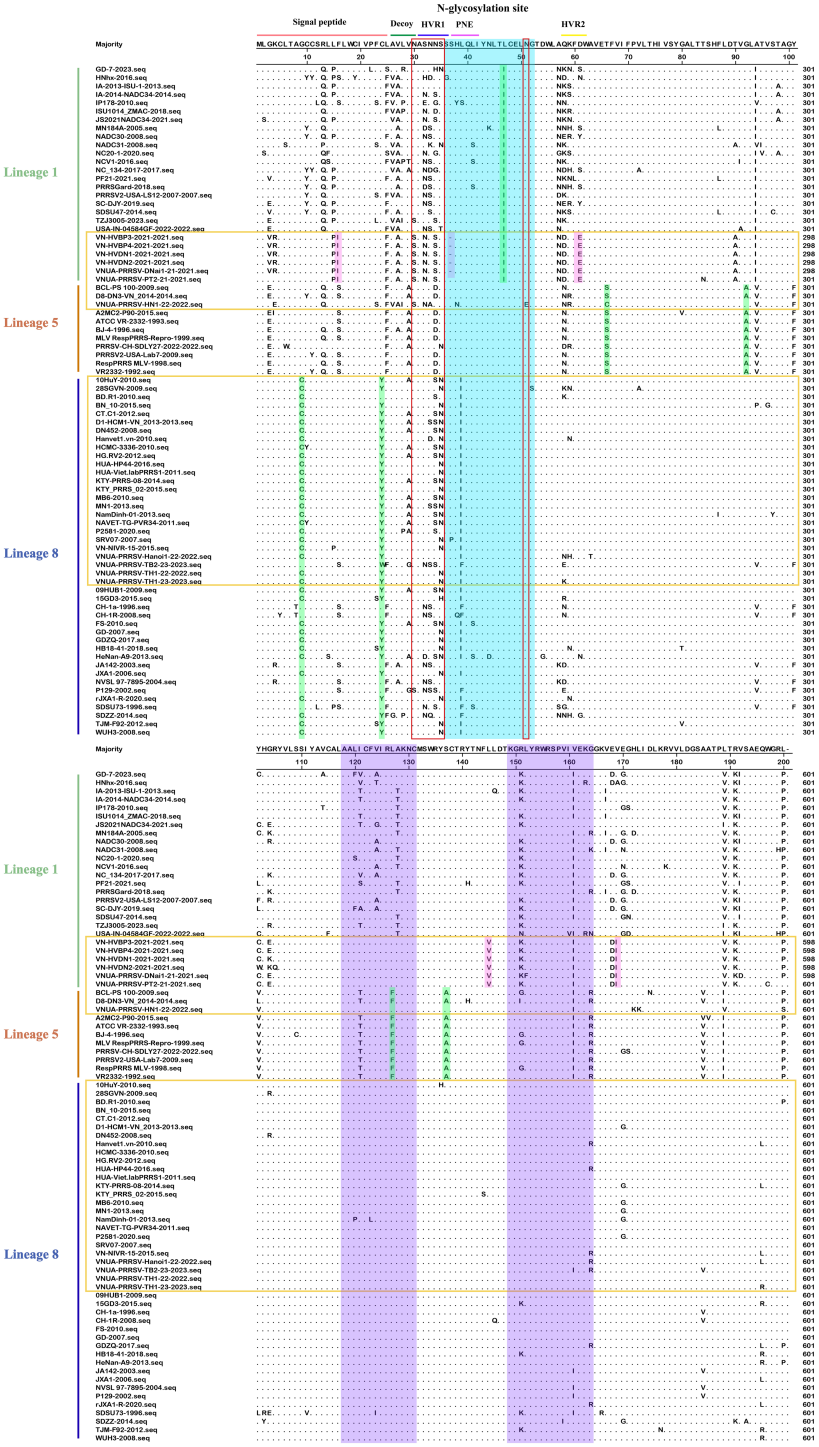
Alignment of 80 PRRSV-2 GP5 amino acid sequences. The Vietnamese strains are marked in yellow. The neutralizing epitope, two T-cell antigenic region, lineage-specific, and Vietnam strain specific mutations are represented by blue, purple, green, and pink, respectively.

Compared with the PRRSV-1 strains, we found that amino acid sites in the PRRSV-2 *GP5* gene appear to be more prone to mutation. These mutations we established to occur mainly in the signal peptide region, decoy epitope, two T-cell antigenic regions, two highly variable regions (HVRs), and in the vicinity of the neutralizing epitope (Figure 7). *N*-glycosylation site mutations were detected at amino acids 30, 32, 33, 34, 35, and 51, mainly in the HVR1 region and neutralizing epitope. Among these, a VNUA-PRRSV-HN1-22-2022 strain (L5A) has the most mutated *N*-glycosylation sites. We identified certain lineage-specific amino acid mutation sites, including a L^47^I mutation in the signal peptide region of GP5 in L1A; G^9^C, and C^24^Y mutations in L8E; and L^93^A, T^66^S, L^127^F, and S^137^A mutations in L5A. In addition, we detected some specific mutations in the L1A strains in Vietnam, including a deletion mutation at the 37^th^ amino acid in the PNE region, and F^26^I, D^61^E, L^145^V, and E^170^I mutations (VNUA-PRRSV-DNAi1-21–2021, VN-HVDN2-2021, VN-HVBP3-2021, VN-HVBP4-2021, VN-HVDN1-2021, and VNUA-PRRSV-PT2-21–2021 strains). Among the three lineages, the strains of L1A and L5A were found to harbor the largest number of strain-specific mutations.

Alignment of the amino acid sequences of the PRRSV-1 and PRRSV-2 GP5 proteins revealed that PRRSV-2 L1A strains are characterized by the largest and most concentrated degree of variation, whereas PRRSV-1 subtype 1 (Global) strains have the lowest degree of amino acid variation. Moreover, compared with the PRRSV-1 strains, we detected a larger number of amino acid mutation sites in the neutralizing and T-cell epitopes of the GP5 sequence in PRRSV-2 strains, thereby indicating that in Vietnam, PRRSV-2 stains are more prone to mutation.

### 3.5 Recombination analysis

Based on multiple alignments of PRRSV GP5 sequences from the 344 PRRSV genomes, we established that there have been no significant recombination events with respect to GP5 protein.

## 4 Discussion

Since the first reported outbreak of HP-PRRS in China in 2006, this disease has spread rapidly throughout other Asian countries, including Vietnam, the Philippines, and Thailand (Do D. T. et al., 2015), and the continuous mutation and recombination of PRRSV present a considerable challenge to the global swine industry (Li et al., 2016). The GP5 protein of this virus, which is essential for virus assembly and infection, and also important for the induction of neutralizing antibodies, is characterized by a notable variability, thereby identifying it as a useful focus for studying the genetic diversity of PRRSV (Jantafong et al., 2015). On the basis of the *GP5* gene of phylogenetic analyses revealed that in recent years, PRRSV-2 lineage 8 strains have been the prevalent strains in Vietnam. The strains of this lineage have been established to be more closely related to lineage 1 strains and most distantly related to those of lineage 5 strains, whereas PRRSV-1 subtype 1 (global) strains were closer to subtype 3 strains and distant from subtype 1 (Russia). A comparison of the evolutionary trees constructed using the ML and NJ methods revealed no differences in the genetic distances between PRRSV-1 and PRRSV-2, or among different PRRSV-2 lineages. This observation may be related to the selected PRRSV strain gene from Vietnam. The emergence of HP-PRRS was first reported in China in 2006, and in the following year, an outbreak of HP-PRRS was reported in northern Vietnam, which subsequently spread rapidly to central and southern regions (Do T. D. et al., 2015; Tian et al., 2007). Between 2008 and 2012, Thuy et al. (2013) isolated 32 PRRSV strains of the PRRSV-2 sublineage 8.7 in Vietnam, a majority of which were found to be closely related to the JXA1-2006 strain isolated from China. It has been proposed that PRRS outbreaks are also associated with climatic factors (Alkhamis et al., 2017). Given the close geographical proximity of northern Vietnam to China, it is speculated that these factors may have contributed to a close correlation in the PRRSV strains transmitted between Vietnam and China. In 2021, PRRSV-2 lineage 1 strains were reported in Vietnam for the first time, along with the isolation of a vaccine-like strain, VN-HVLC1-2021 (belonging to L8E) (Nguyen et al., 2022). Accordingly, it is speculated that there may have been variation or recombination in the PRRSV vaccine strains used on Vietnamese farms. In this regard, the phylogenetic trees constructed in this study reveal that the PRRSV-2 L8E strains are the most abundant among those isolated in Vietnam, whereas L1A strains are the least abundant. Although to date there have been no relevant systematic reports, we found that whereas PRRSV-1 strains were isolated in Vietnam in 2016, PRRSV-2 L1A strains appeared in 2021, the number of L5A strains exhibited a gradual increase in 2022, thereby highlighting the emerging complexity of the epidemiological and genetic evolution of PRRSV on Vietnamese swine farms. Although our analyses in this study were constrained to a certain extent by the limited number and variety of PRRSV sequences obtained from strains isolated in Vietnam, on the basis of the types of PRRSV strains isolated in recent years, it would be predicted that the prevalence of PRRSV-1 strains, along with PRRSV-2 lineage 1 and 5 strains may gradually increase in the future.

In this study, we accordingly assessed 52 PRRSV-1 and 80 PRRSV-2 strains to investigate the nucleotide and amino acid homologies and amino acid sequence alignments of the GP5 protein, which thereby enabled us to establish the genetic diversity of this protein in Vietnam and predict potential evolutionary trends. Our findings revealed nucleotide and amino acid homologies of 76.1–100.0% and 75.2–100.0%, respectively, for PRRSV-1 strain GP5 proteins and corresponding values of 81.8–100.0% and 81.1–100.0% for PRRSV-2 strain GP5 proteins. Among these, PRRSV-1 subtype 1 (Global) exhibited the lowest homology and the most distant phylogenetic relationship with subtype 1 (Russia). Furthermore, we established that the *GP5* gene sequences of the Vietnamese PRRSV-2 strains CT.C1-2012 and HG.RV2-2012 are identical to that of the Chinese PRRSV-2 strain 09HUB1-2009 (100%), whereas we detected no close similarities regarding PRRSV-1 strains, thereby tending to indicate that the evolutionary trends of PRRSV strains in Vietnam and China are both similar and distinct.

*N*-glycosylation sites on the GP5 protein are essential for viral replication and play important roles in receptor binding, viral infectivity, and the induction of immune responses (Wei et al., 2012). It has been shown that different *N*-glycosylation site mutations have differing effects on PRRSV, including loss of polysaccharide residues in N^34^, N^44^, and N^51^ enhancing immunogenicity, whereas those at N^33^, N^34^, and N^35^ may enhance viral virulence; moreover, mutations in neutralizing epitopes can also lead to changes in PRRSV virulence and immune escape (Luo et al., 2023). Furthermore, specific mutations in *N*-glycosylation sites, such as D^37^N, D^56^N, S^60^N, and G^63^N, have been established to be closely associated with immune evasion, inhibition of host viral recognition, and loss of host neutralization capacity (Hsueh et al., 2023). In the present study, we identified ten *N*-glycosylation site mutations in the GP5 protein of Vietnamese PRRSV strains (4 in PRRSV-1, 6 in PRRSV-2), particularly at residues 33, 34, 35 and 51 of the PRRSV-2 GP5 protein, which may potentially play an important role to immune evasion or altered PRRSV virulence. All four glycosylation sites of PRRSV-1 were conserved, with mutations observed in only a few individual strains; whereas among the Vietnamese PRRSV-2 strains, the N^51^ site was relatively conserved, with mutations observed only in the strain VNUA-PRRSV-HN1-22-2022. Guo et al. (2019) have previously found that mutations to the 39^th^ and 57^th^ amino acids of GP5 contribute to virus evasion of host immune responses, and the 187–200 amino acid positions of the GP5 positions in both PRRSV-1 and PRRSV-2 were instrumental in the cross-neutralization reaction of antibody formation (de Lima et al., 2006). Compared with the findings reported by Nguyen et al. (2022), we identified certain identical amino acid mutation sites (aa 39, 52–61, 187–200) in the PRRSV-2 GP5 protein, which may confer the virus with the ability to evade host immunity; and certain different amino acid mutation sites were identified in sublineage L1A, including a novel deletion mutation (aa 37), a lineage-specific mutation (L^47^I) and four mutations specific (F^26^I, D^61^E, L^145^V, and E^170^I). In contrast, among the Vietnamese PRRSV-1 strains, with the exception of EuroViet-01-2016 and EuroViet-02-2016, which exhibited mutations at amino acid positions 187–200, no other strains demonstrated mutations at these sites. Furthermore, two specific mutations were identified in PRRSV-1 subtype 1 (Global): H^5^C and A^201^V. However, the association of these mutations with the epidemiological and genetic variation trends of PRRSV strain in Vietnam will necessitate further investigation.

It has been established that PRRSV spreads between farms via the transfer of infected animals and infected semen, aerosols, and items contaminated with the virus (Lee et al., 2019a), and Zimmerman et al. (1997) found that birds may be involved in the transmission of PRRSV. Accordingly, given that many small-scale farms in Vietnam operate mixed livestock farming systems (Lee et al., 2019b), the potential for virus transmission from avian species to pigs cannot be ruled out. Notably, such small-scale swine farms account for 70% of the total pig production in Vietnam, and farmers rearing pigs on these farms rarely use PRRSV vaccine (Lee et al., 2019a). Consequently, given the inadequate vaccine coverage and lack of effective biosecurity measures, these small-scale farms are at a heightened risk of PRRS outbreaks. Outbreaks are, however, by no means confined to these smallholdings, as PRRS outbreaks still occur on swine farms that have been conducting vaccination over prolonged periods, which would indicate that new mutations and antigen changes in PRRSV strains may have led to immune evasion. In addition, Vietnamese farmers continue to sell sick pigs in order to reduce the economic losses caused by PRRS, thereby making the prevention and control of PRRS even more challenging (Huong et al., 2016).

To summarize, in this study, we sought to examine nucleotide and amino acid homologies among different strains of PRRSV and characterized amino acid mutations in these strains based on analyses of the genetic dynamics of 271 PRRSV GP5 sequences obtained from Vietnamese strains isolated between 2007 to 2023. Although the GP5 protein of this virus has been established to show significant variability and is prone to recombination events, we detected no relevant recombination in this study, and we suspect that the recombination events may exist in other ORFs. During the course of PRRS epidemics, PRRSV strains undergo continuous mutation and evolution, thereby giving rise to numerous novel strains that can compromise the efficacy of vaccines. Moreover, as a consequence of economic imperatives and a lack of biosecurity awareness, small-scale farms in Vietnam tend to be characterized by low PRRSV vaccination rates and unrestricted pig trade, thereby presenting considerable challenges for ensuring effective PRRS prevention and control. On the basis of our analysis of PRRSV GP5 sequences obtained from strains isolated in different years, we provide a theoretical foundation for further research and the development of novel vaccines based on the *GP5* gene.

## 5 Conclusions and outlook

In this study, we established that from 2007 to 2023, the PRRSV-1 strains prevalent in Vietnam were of subtype 1 (Global), along with sublineages L1A, L5A, and L8E PRRSV-2 strains, among which, L8E strains have tended to be the predominant type, followed by L5A, whereas L1A became prevalent only from 2021. Phylogenetic analysis based on sequences of the *GP5* gene enabled us to assess the genetic diversity of PRRSV strains in Vietnam, and using such information, continuous monitoring of PRRSV GP5 genetic variation should be conducted to strengthen PRRSV prevention and control measures in this country.

On the basis of a survey of reports related to PRRSV in Vietnam, it has been established that the outbreaks of PRRS epidemics in the country can largely be traced to small-scale swine farms that account for the highest proportion of Vietnamese pig production. This can be ascribed to the fact that these farms tend to be based on mixed livestock and poultry breeding systems, combined with low vaccination rates against PRRSV and a lack of biosecurity awareness among pig farmers. Nevertheless, even on farms in which livestock have been routinely vaccinated against PRRS, herds still exhibit clinical symptoms related to PRRS, which provides evidence to indicate that PRRSV strains mutate and recombine to evade vaccine-promoted immunity, and thus highlights the inability of vaccines currently administered in Vietnam to effectively prevent and control PRRS. Given that the PRRSV GP5 protein shows high variability, and that mutations in the amino acid sequence can variously influence the virulence and immune evasion of PRRSV, as well as the neutralization capacity of the host, in-depth analyses of trends in the genetic variation of this protein could provide a theoretical basis for the development of novel vaccines and the establishment of effective prevention and control measures. Moreover, it is imperative that Vietnam further strengthens the cultivation of biosafety awareness among small-scale farmers and implement relevant biosafety measures, which, we anticipate, would contribute to the future control or even eradication of PRRS.

## Data Availability

The original contributions presented in the study are included in the article/Supplementary material, further inquiries can be directed to the corresponding authors.

## Footnotes

^1^ https://itol.embl.de
